# The Impairment of TorsinA's Binding to and Interactions With Its Activator: An Atomistic Molecular Dynamics Study of Primary Dystonia

**DOI:** 10.3389/fmolb.2018.00064

**Published:** 2018-07-10

**Authors:** Emmanuel O. Salawu

**Affiliations:** ^1^TIGP Bioinformatics Program, Academia Sinica, Taipei, Taiwan; ^2^Institute of Bioinformatics and Structural Biology, National Tsing Hua University, Hsinchu, Taiwan; ^3^School of Computer Science, University of Hertfordshire, Hertfordshire, United Kingdom; ^4^Bioinformatics Center, Sheridan, WY, United States

**Keywords:** primary dystonia, neurodegenerative disorder, TorsinA, LULL1, glutamic acid, deletion, crystallization agent, mutation

## Abstract

Primary dystonia's prolonged muscle contractions and the associated abnormal postures and twisting movements remain incurable. Genetic mutation/deletion of GAG from TorsonA's gene resulting in ΔE303 (which weakens the binding between TorsinA and its activator, such as LULL1) primarily cause this neurodegenerative disorder. We studied TorsinA-LULL1 (or TorsinAΔE303-LULL1) bindings and interactions. For the first time, we show the atomic details of TorsinA-LULL1 dynamic interactions and TorsinAΔE303-LULL1 dynamic interactions and their binding affinities. Our results show extensive effects of ΔE303 on TorsinAΔE303-LULL1 interactions, and suggest that the differences between TorsinA-LULL1 interactions and TorsinAΔE303-LULL1 interactions are non-subtle. ΔE303 significantly weakens TorsinAΔE303-LULL1's binding affinity. We present pieces of evidence proving that the effects of ΔE303 (on the differences between TorsinA-LULL1 interactions and TorsinAΔE303-LULL1 interactions) are more pronounced than previously suggested, and that the nanobody used for achieving the X-ray crystallization in the previous study attenuated the differences between TorsinA-LULL1 and TorsinAΔE303-LULL1 interactions. Our accounts of the dynamic interactions between “TorsinA and LULL1” and between “TorsinAΔE303 and LULL1” and the detailed effects of ΔE303 on TorsinA-/TorsinAΔE303-LULL1 build on previous findings and offer new insights for *a better understanding of the molecular basis of Primary Dystonia*. Our results have *long-term potentials of guiding the development of medications* for the disease.

## Introduction

**Figure 1:**
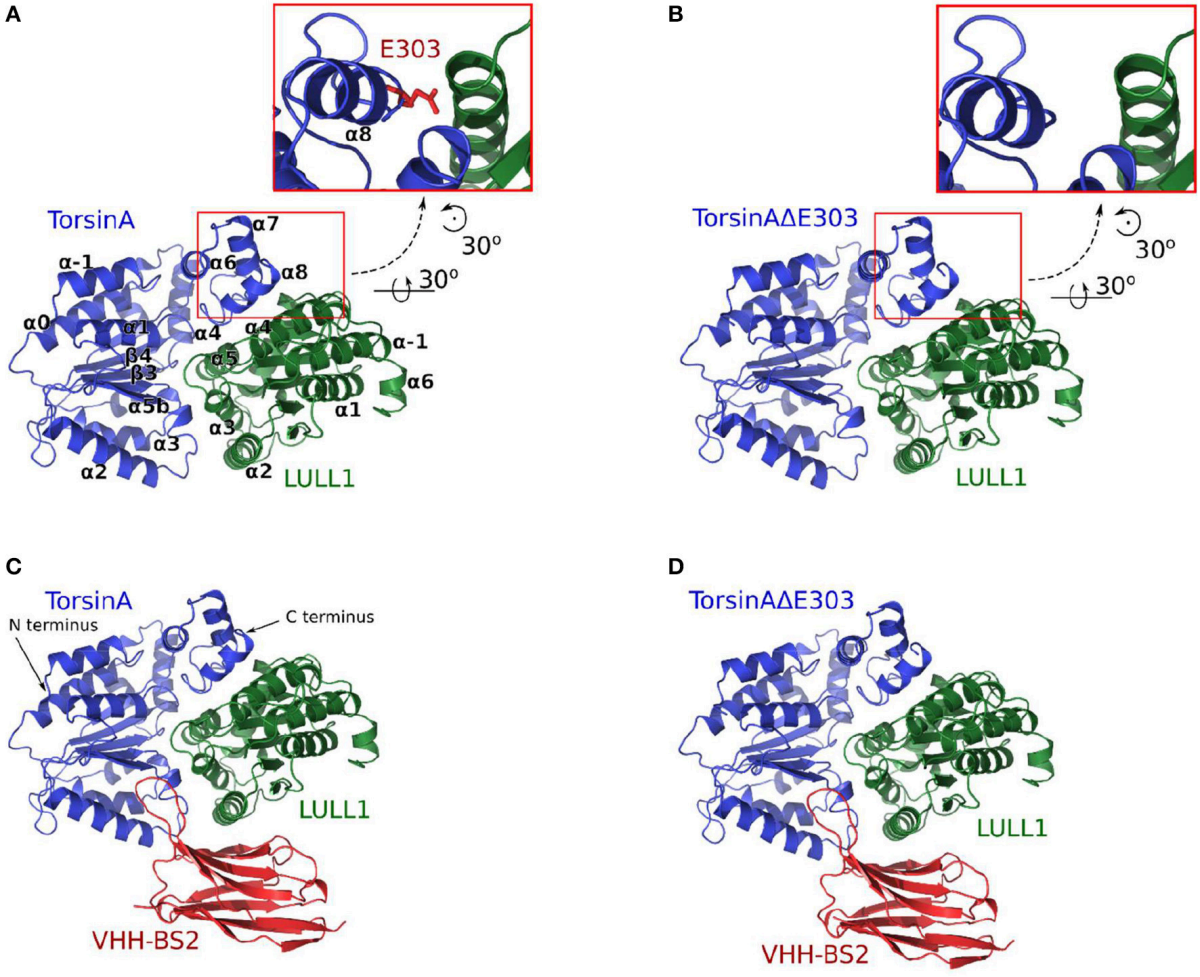
The four molecular systems studied are shown in **(A)** TorsinA+LULL1, **(B)** TorsinAΔE303+LULL1, **(C)** TorsinA+LULL1+VHH-BS2, and **(D)** TorsinAΔE303+LULL1+VHH-BS2. The inset in **(A)** shows the position of E303. VHH-BS2 (shown in **C,D**) is a nanobody used in the crystallization. The atomic coordinates obtained from the nanobody-aided X-ray crystallography have been described in a separate study (Demircioglu et al., 2016) and are in the PDB with accession identifications 5J1S and 5J1T for the wild type and the mutant respectively.

Primary dystonia is a neurodegenerative disease characterized by prolonged involuntary muscle contractions, twisting movements, and abnormal postures (Fahn, 1988; Breakefield et al., 2008; Demircioglu et al., 2016). The disease affects 2-7320 individuals per million people (Defazio et al., 2004) with prevalence varying very widely across populations (Dystonia in Europe (ESDE) Collaborative Group and others, 2000; Müller et al., 2002; Defazio et al., 2004; Steeves et al., 2012). Dystonia as a whole (i.e. primary dystonia, secondary dystonia, and dystonia plus) are essentially motor symptoms of one or more underlying pathophysiological states and could be triggered by a number of insults (Kojovic et al., 2013). Since the symptoms of dystonia resembles those from basal ganglia lesions, researchers initially thought that primary dystonia was a basal ganglia disease (Marsden et al., 1985). Although cerebellar deficit may contribute to primary dystonia's symptoms (Sadnicka et al., 2012), studies have shown that genetic mutation in DYT1 gene (which is often hereditary and seldom sporadic) resulting in the deletion of glutamic acid 303 (hereafter referred to as ΔE303, Figure 1) from TorsinA protein (primarily found in nerve cells) is the most common cause of primary dystonia (Ozelius et al., 1997; Leung et al., 2001; Breakefield et al., 2008. Since primary dystonia results from a dominant allele, a copy of the mutated DYT1 gene is sufficient for the disease to manifest such that a child could have the disease if at least one of his/her parents carry the gene for the disease. However, the genetic defect exhibits incomplete penetrance such that only about 30% of the carriers show clinically detectable symptoms of dystonia (Sharma et al., 2005).

Being a member of the AAA+ ATPase enzymes family, a normal and functional TorsinA (like other family members) essentially hydrolyzes ATP and uses the energy released to drive cellular activities (Hanson and Whiteheart, 2005), such as modification of the structures of other molecules. However, unlike the other self-activating AAA+ ATPase enzymes, TorsinA must bind to and interact with either “**L**amina-**A**ssociated **P**rotein **1** (LAP1)” or “**LU**minal Domain **L**ike **L**AP**1** (LULL1, a paralog of LAP1, Figure 1)” to be activated (Zhao et al., 2013; Brown et al., 2014; Rose et al., 2015). The TorsinA's ΔE303 reduces its binding to (and its interactions with) LULL1 or LAP1. Unable to be properly activated (due to its weak binding to or its dissociation from its activator), the defective TorsinA ultimately causes disruptions in neuronal communications and muscular controls, which results in primary dystonia (Breakefield et al., 2008; Naismith et al., 2009).

The atomic structure of TorsinA and TorsinAΔE303 were recently solved (Demircioglu et al., 2016) by nanobody-aided X-ray crystallography. The observed differences in the structures of TorsinA and TorsinAΔE303 made the researchers believe that there is a subtle difference in TorsinA-LULL1 interactions and TorsinAΔE303-LULL1 interactions. The researchers claimed that “a comparison of these structures shows, in atomic detail, the subtle differences in activator interactions that separate the healthy from the diseased state” (Demircioglu et al., 2016). While it is correct that a subtle difference was observed in the structure of TorsinA and TorsinAΔE303 from the nanobody-aided X-ray crystallography structures, the differences between TorsinA-LULL1 interactions and TorsinAΔE303-LULL1 interactions may not necessarily be subtle in the real biological conditions involving dynamic interactions, which are different from the nanobody-aided X-ray crystallography conditions.

Here, we show that the previous account of the effects of ΔE303 on the differences between TorsinA-LULL1 interactions and TorsinAΔE303-LULL1 interactions may have underestimated the effects of ΔE303, and lack atomic details of the dynamic interactions between TorsinA (or TorsinAΔE303) and LULL1. Using 4.8 μs trajectories from our unbiased explicitly-solvated all-atom molecular dynamics (MD) simulations (of the four systems shown in Figures 1A–D), 3.0 μs of Gaussian accelerated MD (GaMD) simulations, and the results from our binding free energy change calculations using Adaptively Biased MD (ABMD) simulations, we show *for the* first *time* (1) the atomic details of the dynamic interactions between TorsinA (or TorsinAΔE303) and LULL1; (2) their binding free energy change; (3) the extensive effects of ΔE303 on TorsinAΔE303-LULL1 interactions; (4) that the differences between TorsinA-LULL1 interactions and TorsinAΔE303-LULL1 interactions are non-subtle but extensive/prominent; and (5) that the nanobody-aided X-ray crystallization conditions attenuated the differences between TorsinA and TorsinAΔE303; such that the effects of the ΔE303 are more pronounced than previously suggested (by nanobody-aided X-ray crystallography results). Nonetheless, we acknowledge that the previous work (Demircioglu et al., 2016), which makes the atomic structure of TorsinA-LULL1 complex and TorsinAΔE303-LULL1 complex available, remains a remarkable and very important contribution to this field of science, and the current work would be impossible without theirs.

## Results and discussions

Based on our four (A:TorsinA+LULL1, B:TorsinAΔE303+LULL1, C:TorsinA+LULL1+VHH-BS2, and D: TorsinAΔE303+LULL1+VHH-BS2, each with three replicates, Figure 1A) unbiased explicit-solvent molecular dynamics (MD) simulations resulting in 4.8 μs production MD trajectories, our 3.0 μs Gaussian accelerated MD (GaMD) simulations, and our binding free energy calculations (all with AMBER16 Pearlman et al., 1995; Case et al., 2005; Salomon-Ferrer et al., 2013 and ff14SB (Maier et al., 2015 force-fields), it is evident that the deletion of glutamic acid 303 from TorsinA, thus TorsinAΔE303, weakens its binding to LULL1, which is in agreement with the findings of previous studies (Németh, 2002; Sharma et al., 2005) which have shown that the ΔE303 mutation disrupts TorsinA's interaction with its activator (Demircioglu et al., 2016), TorsinA's ATPase activities (Konakova and Pulst, 2005), and TorsinA's ability to form multimeric complex (Pham et al., 2006). Furthermore, our results, as discussed in the following subsections, show that important intermolecular interactions are lost or weakened in TorsinAΔE303-LULL1 complex compared to TorsinA-LULL1 complex; that ΔE303 has more severe effects on TorsinA-/TorsinAΔE303-LULL1 interactions and binding than previously suggested in a nanobody-aided X-ray crystallography study (Demircioglu et al., 2016); and that nanobody-aided X-ray crystallization conditions attenuated the differences between TorsinA and TorsinAΔE303, and the differences between their interactions with LULL1. We provide the details and the respective explanations and discussions in the following subsections.

### The deletion of glutamic acid 303 from TorsinA, thus TorsinAΔE303, weakens its binding to LULL1

We calculated the difference between the binding free energy change for TorsinAΔE303-LULL1 and the wild type, WT, TorsinA-LULL1 (i.e. ΔΔG_ΔE303−WT_, shown in Table 1) using both Adaptively Biased Molecular Dynamics (ABMD) simulations (Huber et al., 1994; Darve and Pohorille, 2001; Wang and Landau, 2001; Babin et al., 2008) and Molecular Mechanics Poisson-Boltzmann Surface Area (Kollman et al., 2000; Wang et al., 2001, 2006) (MM-PBSA). Our results show that TorsinAΔE303 has a weaker binding to and fewer interactions with LULL1 compared to the WT TorsinA. This is in agreement with the results of previous studies (Naismith et al., 2009; Zhu et al., 2010; Zhao et al., 2013) which also show that ΔE303 weakens TorsinAΔE303-LULL1's or TorsinAΔE303-LAP1's binding in comparison to the WT TorsinA. Quantitatively, the results of the binding free energy change calculations (via both ABMD and MM-PBSA) show that ΔE303 weakens the binding of TorsinAΔE303 to LULL1 by a ΔΔG_ΔE303−WT_ ≥ 4.7 ± 1.3 kcal/mol (Table 1), which corresponds to TorsinAΔE303-LULL1 having a binding affinity that is significantly lower than the binding affinity of TorsinA-LULL1 (Table 1). In addition to the agreement between our results and the findings of the previous studies, which showed that the ΔE303 weakens the interactions between TorsinAΔE303 and its activator (Naismith et al., 2009; Zhu et al., 2010; Zhao et al., 2013), our results show a quantitative estimate of the differences between the TorsinAΔE303-LULL1's and TorsinA-LULL1's binding free energy change (Table 1) thereby providing new quantitative insights on the effects of ΔE303 in TorsinAΔE303-LULL1's binding. At this point, we must acknowledge that ABMD is more rigorous than MM-PBSA and that the results from the ABMD (Table 1) are better estimates of the free energy changes than the results from MM-PBSA (Table 1) which has been shown to often overestimates free energy changes (Genheden et al., 2011; Homeyer and Gohlke, 2012).

**Table 1:** Binding Gibbs free energy change, ΔG, for TorsinA-LULL1, and TorsinAΔE303-LULL1.

	**ΔG from ABMD (kcal/mol)**	**ΔG from MM-PBSA (kcal/mol)**
Wild Type TorsinA-LULL1 (ΔG_WT_)	−14.12 ± 1.82	−36.10 ± 1.11
TorsinAΔE303-LULL1 (ΔG_ΔE303_)	−9.45 ± 0.57	−30.87 ± 1.14
Effect of ΔE303 on ΔG: ΔΔG_ΔE303−WT_ (ΔG_ΔE303_-ΔG_WT_)	4.67 ± 1.26^**^	5.23 ± 1.12^**^
Effect of ΔE303 on “K_d_ΔE303_/K_d_WT_”	≅1961.64	≅4869.14

** *The ΔΔG_ΔE303−WT_ is statistically (p < 0.001) larger than 0.0, indicating that the ΔE303 significantly weakens TorsinAΔE303-LULL1 binding*.

Furthermore, the ΔG_ΔE303_ of −9.45 ± 0.57 kcal/mol (Table 1) suggests that ΔE303 may not guarantee complete and spontaneous dissociation of LULL1 from TorsinAΔE303 at all times, even though the ΔE303 significantly weakens the binding of LULL1 to TorsinAΔE303 (ΔΔG_ΔE303−WT_ = 4.67 ± 1.26 kcal/mol, Table 1). We believe that this is the reason why it was possible to obtain the X-ray crystallography structure of LULL1 bound to TorsinAΔE303 (Demircioglu et al., 2016). However, it is also very important to note that binding ΔG involving protein-protein interactions (such as in the current case) are prone to overestimation (Noskov and Lim, 2001; Gohlke et al., 2003; Kiel et al., 2004; Andberg, 2011). Therefore, even though the obtained binding ΔΔG_ΔE303−WT_ from the ABMD (Table 1) could be seen as a reliable estimate of the effects of the mutation in weakening TorsinAΔE303-LULL1 binding as compared to TorsinA-LULL1 binding, the individual binding ΔG (namely ΔG_WT_ and ΔG_ΔE303_, Table 1) may have been overestimated and should be interpreted with caution. The effects of potential overestimation of the binding ΔG involving protein-protein interactions should have canceled out in the relative binding energy change, ΔΔG_ΔE303−WT_, making the interpretations of the ΔΔG_ΔE303−WT_ more reliable than the interpretations of the individual binding ΔG (namely ΔG_WT_ and ΔG_ΔE303_).

### Important intermolecular interactions are lost or weakened in TorsinAΔE303-LULL1 complex

**Figure 2:**
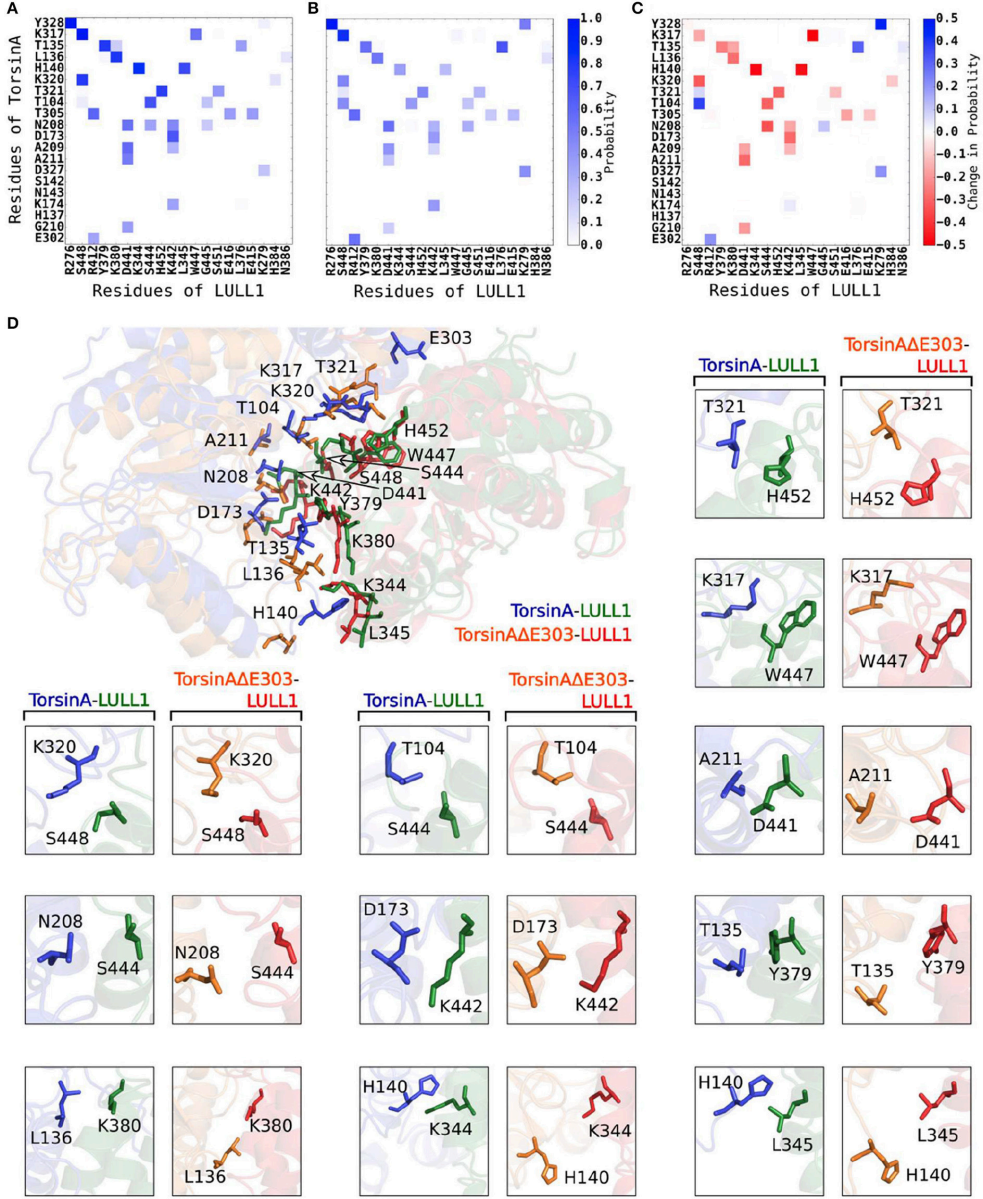
ΔE303 significantly and persistently reduces TorsinAΔE303-LULL1 interactions compared to TorsinA-LULL1 interactions. We show the most active residues **(A)** in TorsinA-LULL1 interactions and **(B)** in TorsinAΔE303-LULL1 interactions, and **(C)** the interactions changes due to ΔE303 (i.e., **B** − **A**). The red cells in C indicate the residue-residue interactions that are lower in TorsinAΔE303-LULL1 compared to TorsinA-LULL1. The details of the weakened TorsinAΔE303-LULL1 interactions as compared to TorsinA-LULL1 interactions from a representative frame from the MD trajectory are shown in **(D)**. The first image in **(D)** show the overall differences in the TorsinAΔE303-LULL1 interactions and the TorsinA-LULL1 interactions. The position of the E303 in the wild type TorsinA (which is deleted from the TorsinAΔE303) is shown at the top of the image. The comparisons of each pair of residues are shown in the rest of panel D with TorsinA-LULL1 interactions shown in blue and green, and TorsinAΔE303-LULL1 interactions shown in orange and red. Only the top 11 most weakened interactions are shown in details.

**Figure 3:**
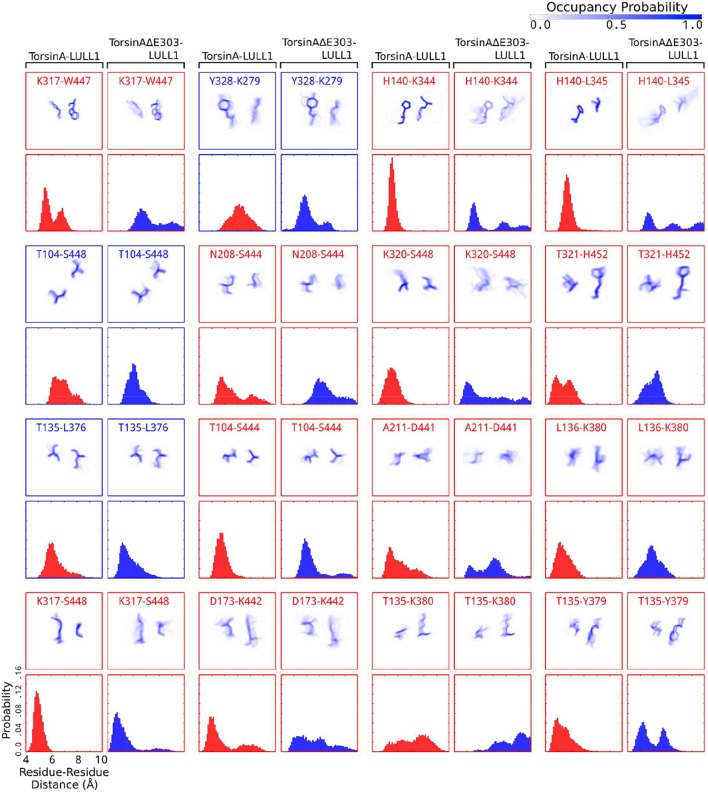
Detailed account of the effects of ΔE303 on the distributions of contacts/distances between TorsinA's residues and LULL1's residues. We show a more detailed account of the effects of ΔE303 on residue-residue *(“amino acid of TorsinA/TorsinA*Δ*E303”–“amino acid of LULL1” e.g., K317-W447)* distances. A red bounding-box shows that (for the given TorsinA-/TorsinAΔE303-LULL1 residue pair) TorsinA-LULL1 interactions are better than TorsinAΔE303-LULL1 based on a cut-off distance of 6 Å, otherwise a blue bounding-box. In each group of four boxes, the top row shows the distributions of the positions of the amino acid of TorsinA-/TorsinAΔE303 and that of LULL1 in form of occupancy probability ranging from 0.0 (white) to 1.0 (dark blue), while the bottom row contains histograms showing the distributions of TorsinA-/TorsinAΔE303-LULL1 distances. The residue pairs average distances were sorted in descending order based on the magnitude of the differences between TorsinA-LULL1 and TorsinAΔE303-LULL1 such that those with the largest absolute differences are listed first. Only 16 pairs are shown in this figure. Additional examples are presented in Supplementary Figure S1.

Using a cut-off distance of 6 Å between residues' centers of mass, we carried out residue-residue contact analysis for both the TorsinA-LULL1 and the TorsinAΔE303-LULL1 molecular systems over the unbiased production MD trajectory. Furthermore, we decomposed the binding enthalpy obtained from the MM-BPSA over the unbiased production MD trajectory into the residue-level contributions so as to be able to assess the contributions of each of the residues toward TorsinA-/ TorsinAΔE303-LULL1 binding. The results from the residue-residue contact analysis and from the MM-PBSA show that Y328, K317, T135, L136, H140, K320, T321, T104, T305, N208, D173, A209, A211, D327, K174, G210, E302, are the amino acids of TorsinA playing the most vital roles in the dynamic interactions between (as well as in the favorable binding of) TorsinA and LULL1 (Figure 2, Supplementary Figure S1). The favorable TorsinA-LULL1 interactions/binding that are mediated by these residues are highly compromised in the mutant, TorsinAΔE303-LULL1 (Figures 2, 3, Supplementary Figure S1) as shown by the red bins in Figure 2C, and longer residue-reside distances in the TorsinAΔE303-LULL1 systems shown in Figures 2D, 3.

Furthermore, we found that the differences between TorsinA-LULL1 interactions and TorsinAΔE303-LULL1 interactions do not involve only a few amino acids pairs but rather involves many amino acids pairs. For example, we observed significant and persistent weakening of the interactions between the following 21 pairs of amino acids of “TorsinAΔE303 and LULL1” compared to the corresponding interactions between the amino acids of “TorsinA and LULL1” sorted in the descending order of the effect of ΔE303 on their interactions: K317&W447, A141&K344, A141&L345, A209&S444, K320&S448, T321&H452, G105&S444, E212&D441, K174&K442, H137&K380, L136&Y379, A211&D441, T305&E416, G210&D441, A209&K442, L136&K380, K317&S448, G210&K442, T321&S451, T305&E415, and K320&H384 (Figures 2, 3, Supplementary Figure S1). However, it appears that the adverse effects of ΔE303 were mildly ameliorated by increases in Y328&K279, G105&S448, L136&L376, D327&K279, and E303&R412 interactions for TorsinAΔE303-LULL1 (Figures 2C, 3, Supplementary Figure S1).

### ΔE303 has more severe effects on TorsinA/TorsinAΔE303-LULL1 interactions and binding than previously suggested

**Figure 4:**
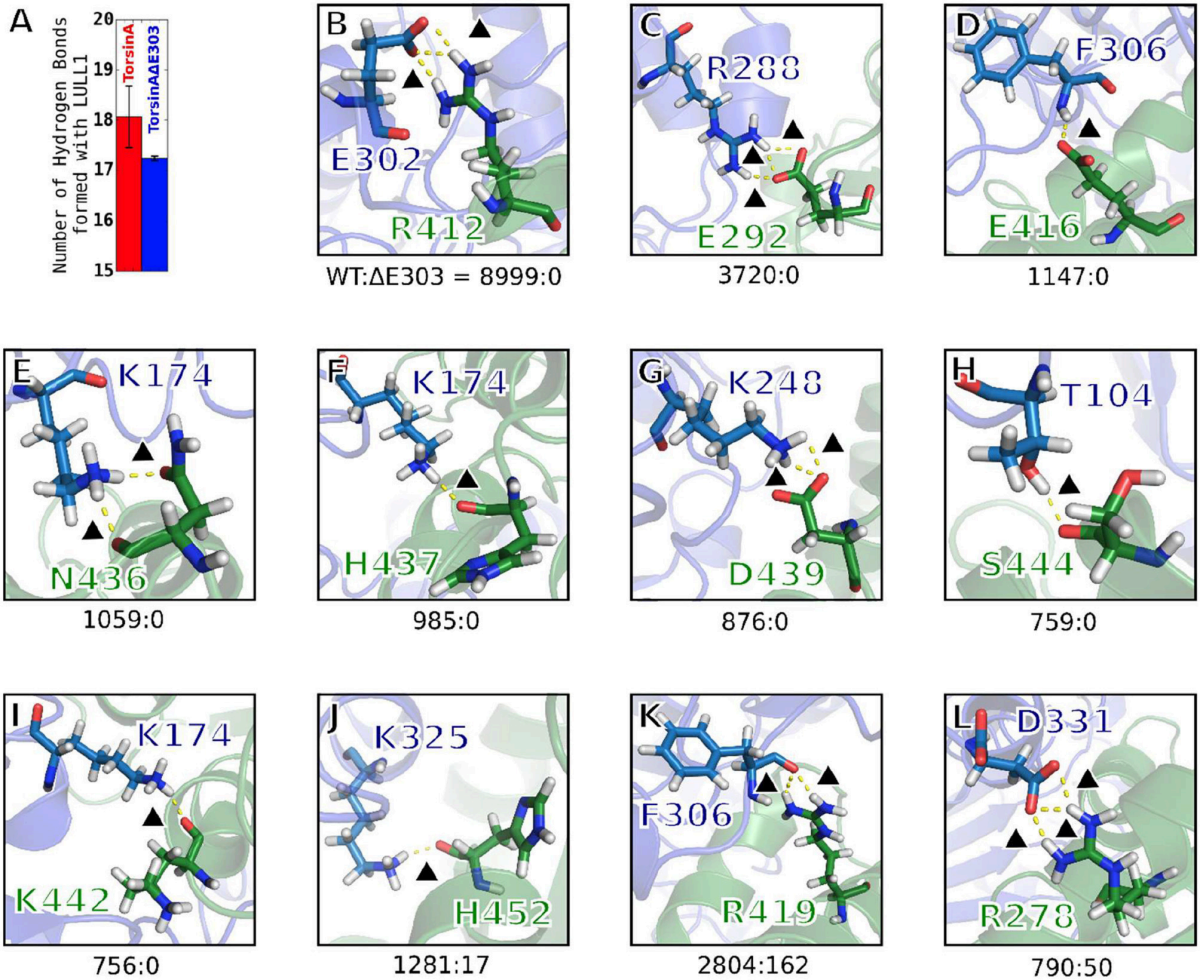
Hydrogen bonding between TorsinA and LULL1 is highly compromised by ΔE303. **(A)** Compared to TorsinA, TorsinAΔE303 forms fewer hydrogen bonds with LULL1. The difference is statistically significant (*p* < 0.001). Panels **(B–L)** show examples of hydrogen bonds that are frequently seen in TorsinA-LULL1 interactions but are never (or seldom) seen in TorsinAΔE303-LULL1 interactions. The hydrogen bonds were assessed in 4,500 frames out of 45,000 frames from the MD trajectories by choosing every tenth frame. Please, note that one frame may contain two or more hydrogen bonds between a given residue pairs, hence the possibility of having the overall hydrogen bonds frequency for a given residue pairs (such as 8,999 for e302-R412) greater than the number of frames. The hydrogen bonds are represented by yellow broken lines. A black triangle is placed next to each of the hydrogen bonds to guide the readers' eyes. The amino acids of TorsinA are shown in blue, while those of LULL1 are shown in green. The ratio of the frequency of a hydrogen bonding pattern in TorsinA-LULL1 interactions to its frequency in TorsinAΔE303-LULL1 interactions are presented as the numbers below each panel (e.g. TorsinA-LULL1:TorsinAΔE303-LULL1 = 8999:0 in panel B). Additional examples are provided in Supplementary Figure S2.

While investigating the TorsinA-/TorsinAΔE303-LULL1 intermolecular interactions, we assessed the differences between TorsinA-LULL1 and TorsinAΔE303-LULL1 hydrogen-bonding frequencies. Our detailed investigations of these basic intermolecular interactions, show significant differences between TorsinA-LULL1 hydrogen bonds frequencies and TorsinAΔE303-LULL1 hydrogen bonds frequencies (Figure 4, Supplementary Figure S2).

We observed that hydrogen bonding between TorsinAΔE303 and LULL1 is compromised by the deletion of glutamic acid 303, ΔE303, in TorsinAΔE303-LULL1. Compared to TorsinA, TorsinAΔE303 forms statistically significant (*p* < 0.001) fewer hydrogen bonds with LULL1 (Figure 4A). Some important TorsinA-LULL1 hydrogen bonds are completely absent (e.g. E302-R412, R288-E292, F306-E416, etc.) or seldom present (e.g. K325-H452, F306-R419, D331-R278, etc.) in TorsinAΔE303-LULL1 interactions (Figures 4B–L, Supplementary Figure S2). In other words, for example, the hydrogen bonds between TorsinAΔE303's E302 and LULL1's R412 are abolished in TorsinAΔE303-LULL1 (Figure 4B), while the hydrogen bonds between TorsinAΔE303's K325 and LULL1's H452 (Figure 4J) are reduced by about 100 folds, etc. These further show that the differences between TorsinA-LULL1 interactions and TorsinAΔE303-LULL1 interactions are not subtle. We think that the previous (Demircioglu et al., 2016) observation of subtle difference may have resulted from the effects of the nanobody used in the nanobody-aided X-ray crystallization of the complex and/or because X-ray crystallography cannot offer information on the dynamic interactions between the proteins. We further explain this in the next section.

It is surprising, but interesting, that the deletion of glutamic acid 303, ΔE303, from TorsinAΔE303-LULL1 would result in the complete loss of the hydrogen bonds between TorsinAΔE303's E302 and LULL1's R412 in TorsinAΔE303-LULL1 (TorsinA:TorsinAΔE303 = 8999:0, Figure 4B), the complete loss of the hydrogen bonds between TorsinAΔE303's R288 and LULL1's E292 in TorsinAΔE303-LULL1 (TorsinA:TorsinAΔE303 = 3720:0, Figure 4C), etc. These results reinforce the findings of previous studies where it was shown that both E302 (Ozelius et al., 1997) and R288 (Zirn et al., 2008) are important for TorsinA's activation and that their deletion or mutation results in weaker bindings of TorsinA to its activators (Ozelius et al., 1997; Zirn et al., 2008). Our findings (Figures 4B–L, Supplementary Figure S2) suggest that ΔE303 has severe effects on TorsinAΔE303-LULL1 interactions, to the extent of harming other residues of TorsinAΔE303 that would normally have favorable interactions with TorsinA's activator in the wild type with no deletion of E303.

Generally, hydrogen bonds often offer strong intermolecular binding forces, and their loss/absence could, in some contexts, quickly degrade intermolecular binding and interactions. Therefore, the compromised hydrogen bonds in TorsinAΔE303-LULL1 complex (Figure 4, Supplementary Figure S2) can considerably explain the weaker binding between TorsinAΔE303 and LULL1 (compared to the binding between TorsinA and LULL1) (Table 1, Figures 2, 3).

### Nanobody-aided X-ray crystallization attenuated the differences between TorsinA and TorsinAΔE303 and the differences between their interactions with LULL1

The well-pronounced differences we have found between TorsinA-LULL1 interactions and TorsinAΔE303-LULL1 interactions (Figures 2–4, Supplementary Figures S1, S2) made us to carry out further investigations on the dynamic behaviors of TorsinA-LULL1 complex and TorsinAΔE303-LULL1 complex but now in a condition that is a closer to that of the previous nanobody-aided X-ray crystallography study (Demircioglu et al., 2016) by including VHH-BS2 in the studied molecular systems. At this point, we must emphasize that the new molecular system (wherein the VHH-BS2 nanobody is in complex with TorsinA-LULL1 or TorsinAΔE303-LULL1) is essentially a less physiological condition than our original molecular systems' setup (without the nanobody) because TorsinA-LULL1 or TorsinAΔE303-LULL1 is not in complex with VHH-BS2 in the real biological environment. However, this setup allowed us to investigate the potential influence of the nanobody (i.e., VHH-BS2 used in the X-ray crystallography) on the TorsinA-/TorsinAΔE303-LULL1 interactions.

**Figure 5:**
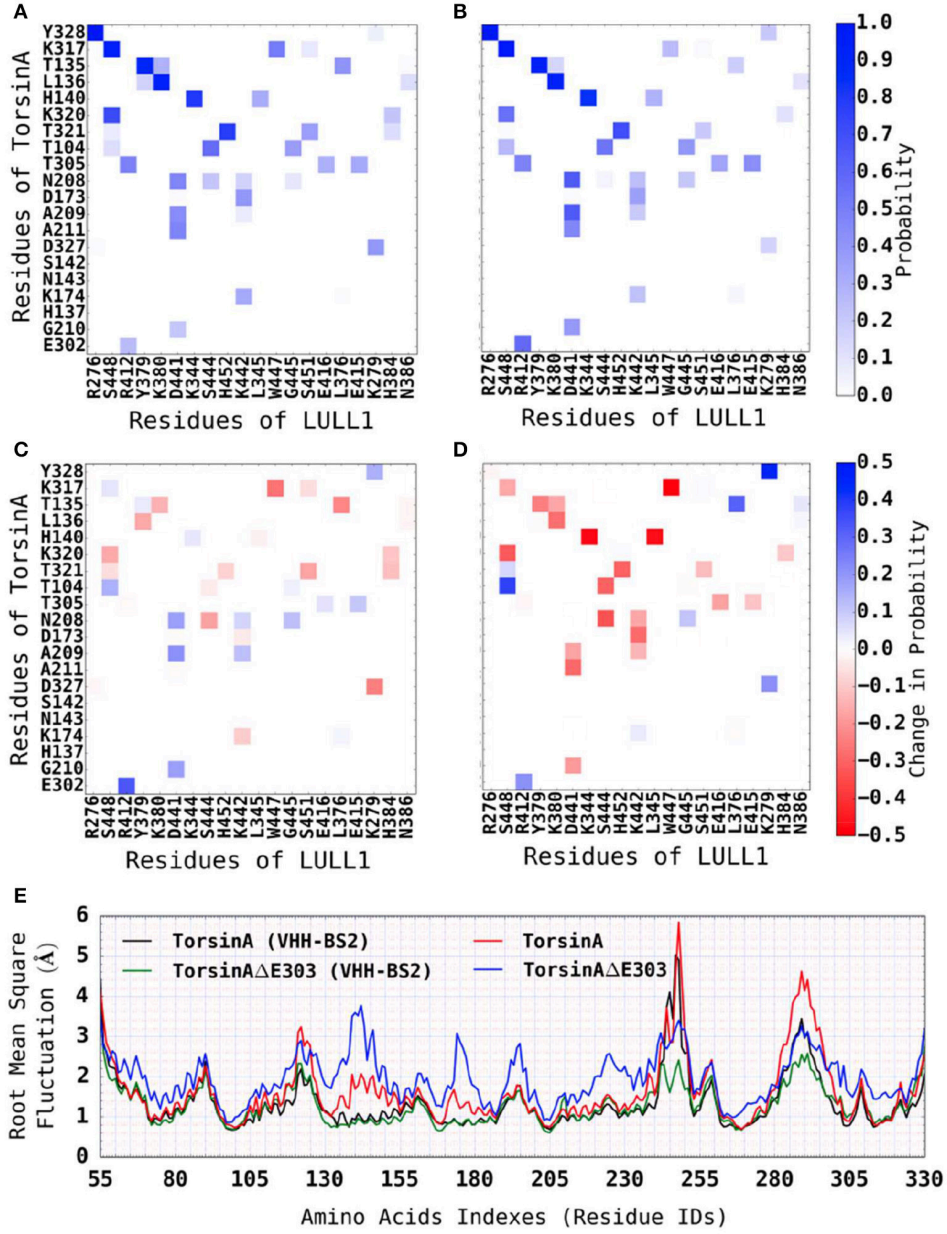
Reduced differences between TorsinA-LULL1 interactions and TorsinAΔE303-LULL1 interactions in the presence of VHH-BS2 nanobody. We show the most active residues **(A)** in TorsinA-LULL1 interactions and **(B)** in TorsinAΔE303-LULL1 interactions, and **(C)** the reduced interactions (i.e., B-A) due to ΔE303. **(D)** Shows similar information as C, but for the TorsinA-/TorsinAΔE303-LULL1 system without VHH-BS2. Additional pieces of evidence that the crystallization conditions (e.g., the presence of VHH-BS2) reduce the difference between TorsinA and TorsinAΔE303 is shown in **(E)** wherein the differences in the RMSF between the amino acids of TorsinA and the amino acids of TorsinAΔE303 is attenuated by the presence of VHH-BS2. The E303 deletion from TorsinA (thus TorsinAΔE303) significantly and persistently reduces TorsinAΔE303-LULL1 interactions compared to TorsinA-LULL1, but the effect of the mutation is attenuated by the crystallization environment/conditions (such as the presence of VHH-BS2). A comparison of **(C,D)** shows (by the fainter shades of blue and red in the current **(C)**, but the darker shades in **D**) that the environment/conditions wherein the X-ray structures for TorsinA-LULL1 and TorsinAΔE303-LULL1 were solved (such as the presence of VHH-BS2) attenuated the differences between TorsinA-LULL1 interactions and TorsinAΔE303-LULL1 interactions.

We observed that the addition of VHH-BS2 to the molecular systems reduced the differences between TorsinA-LULL1 interactions (Figure 5A) and TorsinAΔE303-LULL1 interactions (Figure 5B) as compared to their differences in the absence of VHH-BS2 (i.e., the light red bins in Figure 5C compared to the dark red bins in Figure 5D). These findings (regarding the reduction in the differences between TorsinA-LULL1 and TorsinAΔE303-LULL1 systems in the presence of VHH-BS2) are consistent when we assessed the Root Mean Square (RMS) deviations from the X-ray structure (*not shown, because they are comparable to those from the energy-minimized structure*) or from the energy-minimized structure *{TorsinA: 2.168* ± *0.004; TorsinA*Δ*E303: 2.634* ± *0.009; TorsinA(VHH-BS2): 1.756* ± *0.004Å; TorsinA*Δ*E303(VHH-BS2): 1.772* ± *0.006}*, the RMS fluctuations (Figure 5E), and the secondary structure elements (by RaFoSA Salawu, 2016, Supplementary Table S1) of TorsinA and TorsinAΔE303 in the presence and in the absence of VHH-BS2. Each of these sets of results show that the X-ray crystallization conditions (e.g., the present of the VHH-BS2 nanobody used in the nanobody-aided X-ray crystallography) has the potentials of attenuating the differences between TorsinA and TorsinAΔE303 and the differences between their interactions with LULL1. The RMS deviations and RMS fluctuations essentially reflect the flexibility of a molecular system with larger values signifying more change/flexibility. On the other hand, for a given molecular system, higher proportions of sheets and helixes and lower proportion of coils or loops may indicate less flexibility (Salawu, 2016).

Furthermore, we investigated the effects of the nanobody, VHH-BS2, on the differences between the hydrogen bonding patterns in TorsinA-LULL1 complex and TorsinAΔE303-LULL1 complex. Our comparison of the two complexes in the presence and in the absence of the VHH-BS2 shows that, VHH-BS2 makes TorsinAΔE303-LULL1 unnecessarily stable and makes both Torsin-LULL1 complex and TorsinAΔE303-LULL1 complex to have a few new TorsinA-/TorsinAΔE303-LULL1 hydrogen bonds (such as between D277 and S279, H426 and K266, etc.) that were not present in the absence of VHH-BS2 (Table 2). The effects of VHH-BS2 on TorsinA-/TorsinAΔE303-LULL1 hydrogen bonds is easier to assess by comparing the ratio of the hydrogen bonds in the presence and in the absence of VHH-BS2 as shown in Table 2 as HbR1 compared to HbR2. The hydrogen bonds ratio 1, HbR1, is obtained by dividing the number of hydrogen bonds in TorsinAΔE303-LULL1 by the number of hydrogen bonds in TorsinA-LULL1 for the molecular systems with VHH-BS2. HbR2 is obtained in a similar way but from the molecular systems without VHH-BS2. For a given pair of residues, a value of HbR1 (or HbR2) greater than 1.0 signifies that there are more hydrogen bonds in TorsinAΔE303-LULL1 complex than in the TorsinA-LULL1 complex for that pair of residues (Table 2). The comparison of HbR1 and HbR2 helps in objectively assessing the effects of VHH-BS2 on TorsinA-/TorsinAΔE303-LULL1 hydrogen bonds such that HbR1 > HbR2 suggests that TorsinAΔE303-LULL1 complex has unreasonably high hydrogen bonds relative to TorsinA-LULL1 complex in the presence of VHH-BS2 than in the absence of VHH-BS2, which is the case for a number of the residue pairs shown in Tables 2. This may explain why the weakening of TorsinAΔE303-LULL1 interactions appears to be attenuated in the presence of VHH-BS2.

**Table 2:** In the presence of VHH-BS2 nanobody, TorsinAΔE303-LULL1 complex has atypically high number of hydrogen bonds and show atypical stability^a^.

**Residue Pairs**	**With the nanobody, VHH-BS2**			**Without the nanobody, VHH-BS2**			**Comparison: VHH-BS2 causes unnecessary stability in TorsinAΔE303-LULL1 (i.e., HbR1>HbR2)^c^**
	**TorsinA + LULL1 + VHH-BS2 (TLV)**	**TorsinAΔE303+ LULL1+VHH-BS2 (TΔELV)**	**TΔELV/TLV (HbR1)^b^**	**TorsinA + LULL1 (TL)**	**TorsinAΔE303 + LULL1 (TΔEL)**	**TΔEL/TL (HbR2)^b^**	
D277-S279	13	37	2.85	0	0	0	Yes
D262-W489	28	57	2.04	48	18	0.37	Yes
E427-K266	28	56	2	56	61	1.09	Yes
H426-K266	10	20	2	0	0	0	Yes
E458-K255	17	33	1.94	32	37	1.16	Yes
R461-D262	94	108	1.15	145	93	0.64	Yes
D462-K255	47	52	1.11	53	75	1.42	No
R258-E458	160	170	1.06	240	216	0.9	Yes
D273-K325	66	69	1.05	97	106	1.09	No
R318-E249	116	118	1.02	246	224	0.91	Yes
K263-S493	33	33	1	38	61	1.61	No
E457-K263	79	74	0.937	124	116	0.935	Yes
K321-Y276	21	19	0.9	22	11	0.5	Yes
R454-M250	35	24	0.69	58	42	0.72	No
D277-K321	56	38	0.68	81	90	1.11	No
D277-K325	33	22	0.67	43	32	0.74	No
D273-K321	44	24	0.55	47	44	0.94	No
R454-E249	46	18	0.39	0	0	0	Yes
E457-K271	27	9	0.33	30	30	1	No
H494-T270	21	7	0.33	37	5	0.14	Yes
R461-F252	61	16	0.26	56	3	0.05	Yes
D425-K266	13	3	0.23	0	0	0	Yes
R611-E398	183	0	0	0	0	0	-
R377-D574	164	0	0	0	0	0	-
R454-E248	94	0	0	180	0	0	-
D390-H86	79	0	0	107	0	0	-
G617-H83	65	0	0	0	0	0	-
S88-T565	57	0	0	0	0	0	-
R491-D116	48	0	0	67	0	0	-
T384-T571	42	0	0	0	0	0	-

a *The effects of VHH-BS2 on TorsinA-/TorsinAΔE303-LULL1 hydrogen bonds is assessed by comparing the ratio of the hydrogen bonds in the presence (HbR1) and in the absence (HbR2) of VHH-BS2*.

b *For a given pair of residues, a value of HbR1 (or HbR2) greater than 1.0 signifies that there are more hydrogen bonds in TorsinAΔE303-LULL1 complex than in the TorsinA-LULL1 complex for that pair of residues*.

c *HbR1 > HbR2 suggests that TorsinAΔE303-LULL1 complex has unreasonably high hydrogen bonds relative to TorsinA-LULL1 complex in the presence of VHH-BS2 than in the absence of VHH-BS2*.

**Figure 6:**
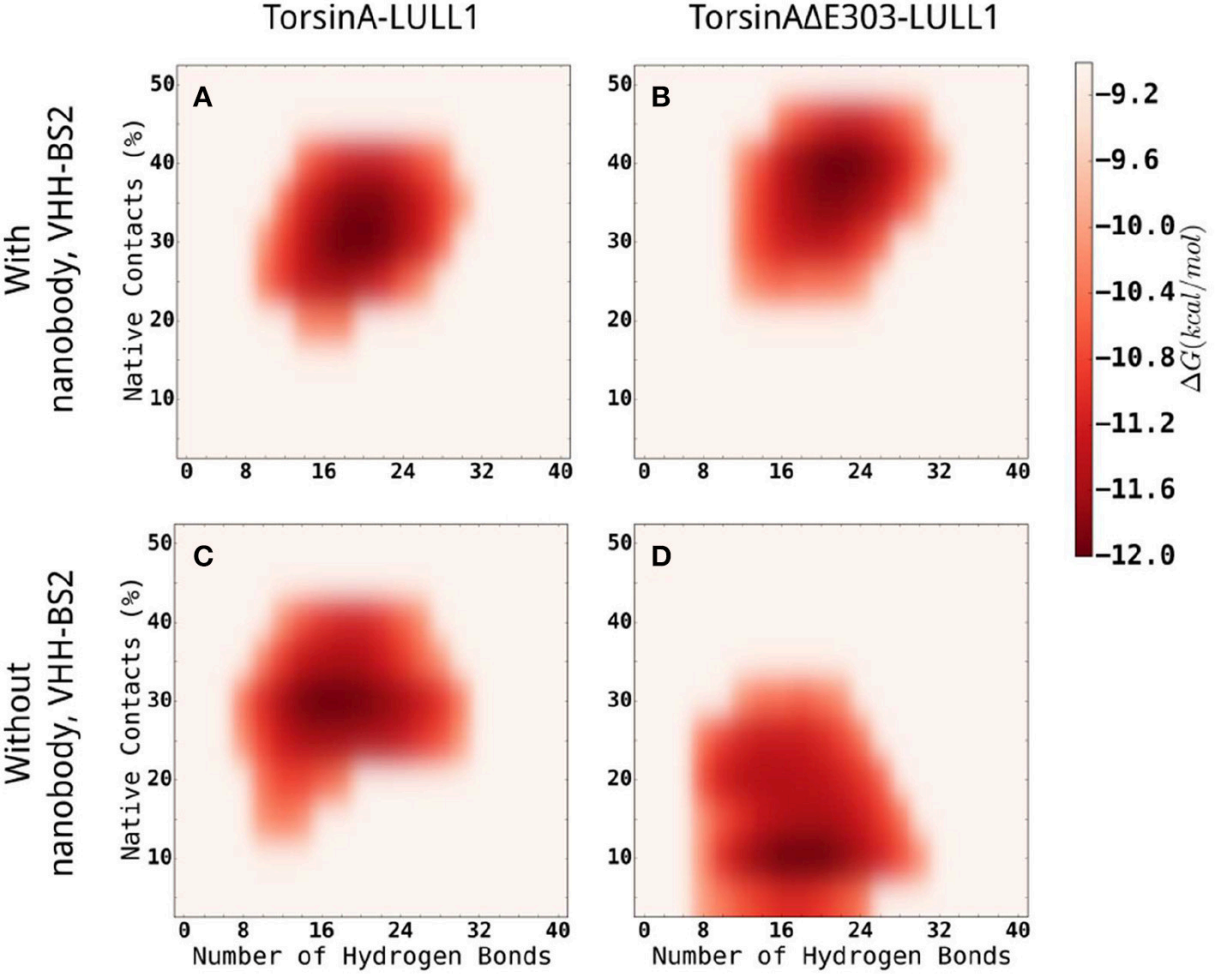
Comparisons of the energy landscapes of the interactions between TorsinA-LULL1 (left column) and TorsinAΔE303-LULL1 (right column) in the presence (top row) of and in the absence (bottom row) of VHH-BS2 nanobody. The energy landscapes were obtained from accelerated sampling using Gaussian accelerated Molecular Dynamics (GaMD) simulations. The energy profiles in **(A,B)** are very comparable because VHH-BS2 attenuated the difference between TorsinA-LULL1 interactions and TorsinAΔE303-LULL1 interactions. The true difference between TorsinA-LULL1 interactions and TorsinAΔE303-LULL1 interactions are more evident in **(C,D)** in the absence of VHH-BS2.

In addition, we carried out enhanced sampling for TorsinA-/TorsinAΔE303-LULL1 complexes with or without VHH-BS2 using Gaussian accelerated Molecular Dynamics (GaMD) simulations (Miao et al., 2015) and constructed the energy landscape for each of the molecular systems studied using TorsinA-/TorsinAΔE303-LULL1's native contacts and TorsinA-/TorsinAΔE303-LULL1's hydrogen bonds as the reaction coordinates (Figure 6). The results show that VHH-BS2 attenuated the difference between TorsinA-LULL1 interactions and TorsinAΔE303-LULL1 interactions. This is evident from the comparable energy profiles in Figures 6A,B. On the other hand, the true difference between TorsinA-LULL1 interactions and TorsinAΔE303-LULL1 interactions becomes more observable in the absence of VHH-BS2 as shown in Figures 6C,D.

From the foregoing, it is apparent that the attenuation of TorsinA-TorsinAΔE303 differences by the nanobody-aided X-ray crystallization conditions (such as the co-crystallization of VHH-BS2 with TorsinA-/TorsinAΔE303- LULL1 complex) might have led to the conclusion of the previous study on the subtle nature of the differences between the healthy TorsinA from the diseased TorsinAΔE303 (Demircioglu et al., 2016). Our results suggest that the nanobody-aided crystallization condition for the X-ray crystallography could not allow the capturing of all the essential interactions between TorsinA (or TorsinAΔE303) and LULL1, and more importantly, the biologically-relevant dynamic interactions between the molecules could not possibly be accounted for, correctly and/or completely, by the X-ray crystallography's results alone as the conditions necessary for the crystallization and/or the presence of the nanobody stripped off some of the proteins' true biological properties and virtually all of their dynamic behaviors. In general, it may be very important that one exercises cautions when making biological/functional interpretations of structural data whenever the crystallization conditions (such as in some nanobody-aided X-ray crystallography) differ sharply from biological conditions because such sharp contractions in the conditions may influence the true biophysical and biochemical nature of the molecule of interest and the true intermolecular interactions between molecular components of the molecular complex of interest.

We acknowledge that a small structural change may signify a considerable functional change or a function loss and that this possibly has motivated structural biology studies. Nonetheless, it is important to recognize the potential limitations of the structural methods such as the possibility that the crystallization environment (for instance, the use of VHH-BS2 nanobody in the current example) may not accurately reflect the biological environment of the molecule(s) of interest and that such difference may affect the observed properties of the molecule(s) of interest, in addition to the inability to observe the dynamic behaviors of the molecule(s). We suggest that the observations from this study regarding the effects of the nanobody on the proteins of interest and their interactions be further investigated, for example on other protein-protein systems, protein-ligand systems, etc. whose structures are only solvable with nanobody-aided crystallography and would not crystalize in the absence of the nanobodies.

## Conclusions

Genetic mutations resulting in the deletion of glutamic acid 303, ΔE303, from TorsinA (thereby weakening the binding between TorsinA and its activator, such as LULL1) primarily cause the (currently) incurable primary dystonia which is a neurodegenerative disorder characterized by prolonged muscle contractions, abnormal postures, and twisting movements. Building on previous works, we have shown the atomic details of TorsinA-LULL1 dynamic interactions and TorsinAΔE303-LULL1 dynamic interactions and their binding free energy changes/binding affinities. Our account of the quantitative value of how much the ΔE303 weakens the binding of TorsinAΔE303 to LULL1 as compared to the binding of TorsinA to LULL1 (ΔΔG_ΔE303−WT_) has never been previously documented and constitutes one of the important contributions of this study to this field. Furthermore, we have presented a detailed account of TorsinAΔE303-LULL1 residue-residue interactions that are compromised by the ΔE303. The presented extensive effects of ΔE303 on TorsinAΔE303-LULL1 interactions show that the differences between TorsinA-LULL1 interactions and TorsinAΔE303-LULL1 interactions are non-subtle but well-pronounced. The complete loss or weakening of vital hydrogen bonds in TorsinAΔE303-LULL1 interactions considerably explains the weakening of the TorsinAΔE303-LULL1 binding (compared to TorsinAΔE303-LULL1 binding). We have also shown that nanobody-aided X-ray crystallization environment in the previous study attenuated the differences between TorsinA-LULL1 and TorsinAΔE303-LULL1 interactions. Pieces of evidence leading to the conclusion that the effects of ΔE303 (on the differences between TorsinAΔE303-LULL1 interactions and TorsinA-LULL1 interactions) are more pronounced than previously suggested have also been presented. Overall, our accounts of the dynamic interactions between “TorsinA and LULL1” and between “TorsinAΔE303 and LULL1” *(such as, but not limited to, the patterns and the frequencies of hydrogen bonds in TorsinA-/TorsinA*Δ*E303-LULL1 complexes)* offer new insights for *a better understanding of the molecular basis of Primary Dystonia* and have *long-term potentials of guiding the development of medications* for the disease.

## Materials and methods

### Structure of TorsinA/TorsinAΔE303-LULL1 complex

We obtained X-ray crystallographic structures of TorsinA/TorsinAΔE303 and its activator (LULL1) in complex with the nanobody (VHH-BS2) from the Protein Data Bank (Berman et al., 2000), PDB IDs: 5j1s, 5j1t (Demircioglu et al., 2016).

### Creation of the initial molecular systems

Using tLeap/AmberTools16 (Pearlman et al., 1995; Case et al., 2005; Salomon-Ferrer et al., 2013) and ff14SB (Maier et al., 2015) force-fields, we created four explicitly solvated initial molecular systems for Molecular Dynamics (MD) simulations with AMBER16 (Pearlman et al., 1995; Case et al., 2005; Salomon-Ferrer et al., 2013): (A) TorsinA+LULL1, (B) TorsinAΔE303+LULL1, (C) TorsinA+LULL1+VHH-BS2, and (D) TorsinAΔE303+LULL1+VHH-BS2 (Figure 1). *VHH-BS2 is a nanobody co-crystallized with TorsinA-LULL1 complex and TorsinA*Δ*E303-LULL1 complex. The crystallization was not possible without VHH-BS2* (Demircioglu et al., 2016).

### Energy minimization

Each of the systems was energy-minimized using AMBER16 (Pearlman et al., 1995; Case et al., 2005; Salomon-Ferrer et al., 2013). The energy minimizations were done in three stages – weakly (0.5 kcal/mol/Å^2^) restraining all non-water atoms in the first stage, all alpha carbon atoms in the second stage, and without any restraints in the third stage. With weak restraints (0.5 kcal/mol/Å^2^) on alpha carbon atoms, each of the systems was gradually heated to 310K in canonical ensemble.

### Molecular dynamics (MD) simulations

The systems' temperatures (Langevin thermostat Pastor et al., 1988 with a collision frequency of 2 ps^−1^) and pressures (Berendsen barostat Berendsen et al., 1984) were controlled during the equilibration and production runs. Full electrostatic interactions energies were calculated using Particle Mesh Ewald method (Darden et al., 1993). A cutoff distance of 10 Å and a cubic spline switch function were used when calculating nonbonded interactions.

Overall, more than 8 μs of all-atom explicit solvent MD simulations were performed. Out of the entire MD simulations performed, 4.8 μs are unbiased production MD–TorsinA+LULL1: 500 ns ^*^ 3 replicates; TorsinAΔE303+LULL1: 500 ns ^*^ 3; TorsinA+LULL1+VHH-BS2: 300 ns ^*^ 3; and TorsinAΔE303+LULL1+VHH-BS2: 300 ns ^*^ 3. Thus, 300 ns ^*^3 + 300 ns^*^3 + 500 ns^*^3 + 500 ns^*^3 = 4,800 ns = 4.8 μs. Gaussian accelerated MD (GaMD) simulations (Miao et al., 2015) for enhanced sampling made up 3.0 μs (i.e., 750 ns for each of the four systems), and Adaptively Biased MD (ABMD) simulations(Babin et al., 2008) made up 240 ns (120 ns for each of “TorsinA + LULL1” and “TorsinAΔE303 + LULL1” systems).

### Binding free energy change calculations by ABMD

Binding free energy change calculations were done using both Adaptively Biased Molecular Dynamics (ABMD) simulations (Huber et al., 1994; Darve and Pohorille, 2001; Wang and Landau, 2001; Babin et al., 2008) and Molecular Mechanics Poisson-Boltzmann Surface Area (Kollman et al., 2000; Wang et al., 2001, 2006) (MM-PBSA).

**Figure 7:**
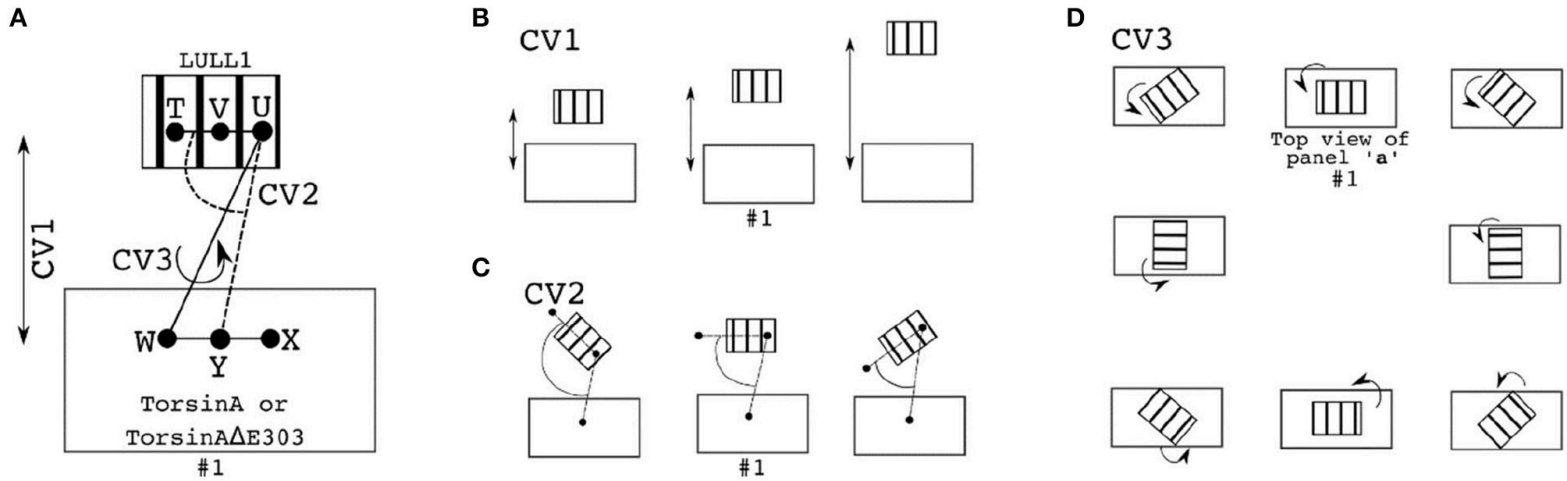
Reaction coordinates for calculating binding Gibbs free energy change through ABMD. In each of the panels/sub-figures, the smaller stripped structure represents the ligand, while the bigger unstripped structure represents the receptor. **(A)** The three reaction coordinates/collective variables (CV) used to define three pairs of CVs are shown as CV1, CV2, and CV3. CV1 is the distance between V (which is the center of mass, CoM, of T and U) and Y (which is the CoM of W and X). CV2 is the angle formed by T, U, and Y, while CV3 is the torsion angle formed by T, U, W, and X. Examples of changes in the position of the ligand relative to the receptor when CV1 is explored/sampled is shown in **(B)**; when CV2 is explored is shown in **(C)**; when CV3 is explored is shown in **(D)**. **D** is made up by views from the top so as to easily demonstrate the effects of changes in the torsional angle (CV3) on the relative orientations of the ligand and the receptor. In each of the **(A–D)**, the setup numbered “#1” depicts the initial setup.

For the adaptively biased molecular dynamics (ABMD) simulations (Huber et al., 1994; Darve and Pohorille, 2001; Wang and Landau, 2001; Babin et al., 2008), three sets of reaction coordinates/collective variables (CVs, Figure 7A) were used. They are (1) CV1 and CV2, (2) CV1 and CV3, and (3) CV2, and CV3. Where CV1 is a distance-based reaction coordinate (Figures 7A,B), CV2 is an angle-based reaction coordinate (Figures 7A,C), and CV3 is a torsion-angle-based reaction coordinate (Figures 7A,D). Each of the ABMD simulations was run until the observed Gibbs free energy change converged.

### Binding free energy change calculations by MM-PBSA

In addition to using ABMD, we calculated the binding free energy for each frame from the production MD simulations trajectory using Molecular Mechanics Poisson-Boltzmann Surface Area (MM-PBSA) (Kollman et al., 2000; Wang et al., 2001, 2006). Although less rigorous than our ABMD approach, the MM-PBSA method we used is advantageous in allowing us to be able to isolate the contributions of each residue to the overall binding energy change. Analytical linearized Poisson-Boltzmann model (Luo et al., 2002; Sigalov et al., 2005, 2006; Wang and Luo, 2010) with the default parameters (except otherwise stated) was used through the MMPBSA.py (Miller et al., 2012) implemented for AmberTools16. The atomic radii specified in the ff14SB force-fields (Maier et al., 2015) and in the ion parameters for Ewald and TIP3P water (Joung and Cheatham III, 2008) were automatically read from the parameter and topology file. The solvent probe radius was also based on the force-fields parameters (Joung and Cheatham III, 2008; Maier et al., 2015). A surface tension of 0.0072 kcal/mol/Å^2^ was used for calculating the nonpolar contribution to solvation free energy. An ionic strength of 0.150 nM was used for the PB solvent.

The binding free energy (Δ*G*_*binding*_) accompanying the complexation of a receptor and a ligand to form a complex can be estimated from MM-PBSA as

(1) $$\begin{array}{l} \Delta G_{binding}=\Delta H-T\Delta S\approx \Delta E_{MM}+\Delta G_{sol}-T\Delta \end{array}$$

where ΔE_*MM*_, Δ*G*_*sol*_ and –*T*Δ*S* are respectively gas-phase Molecular Mechanics (MM) energy change, solvation free energy change, and conformational entropy change upon binding. While –*T*Δ*S* can be computed by normal-mode analysis, ΔE_MM_ and ΔG_sol_ can be expressed as follows.

(2) $$\begin{array}{l} \Delta E_{\text{MM}}=\Delta E_{\text{internal}}+\Delta E_{\text{electrostatics}}+\Delta E_{\text{vdw}} \end{array}$$

(3) $$\begin{array}{l} \Delta G_{\text{sol}}=\Delta G_{\text{PB}}+\Delta G_{\text{SA}} \end{array}$$

where ΔG_*PB*_ is (the polar contribution to) electrostatic solvation energy calculated using Poisson Boltzmann (PB) model. Δ*G*_*SA*_ is (the non-electrostatic contributions to) the solvation component estimated by solvent accessible surface area.

### Enhanced sampling by gaussian accelerated MD (GaMD) simulations

By adding harmonic boost potentials that smoothen the potential energy surface of a molecular system of interest, GaMD enhances the sampling of the conformational space of the molecular system (Miao et al., 2015). Given a molecular system with N atoms, such that the atomic coordinates is $r^{3^{*}N}=r_{1}^{3},r_{2}^{3},\;\ldots ,\;r_{N}^{3}$, the GaMD algorithm modifies the molecular system's potential energy, $V_{original}\left(r^{3^{*}N}\right)$, to $V_{updated}\left(r^{3^{*}N}\right)$ by adding a boost potential, $V_{boost}\left(r^{3*N}\right)$, whenever the original system's potential, $V_{original}\left(r^{3^{*}N}\right)$, is lower than a given threshold, E_*threshold*_. Mathematically,

(4) $$V_{updated}(r^{3\;*\;N})=\{\begin{array}{ll} V_{original}(r^{3\;*\;N})+V_{boost}(r^{3\;*\;N}) & if\;V_{original}(r^{3\;*\;N})\\ \; & <E_{threshold}\\ V_{original}(r^{3\;*\;N}) & else \end{array}$$

where

(5) $$\begin{array}{l} V_{boost}\left(r^{3*N}\right)=\frac{1}{2}k{\left(E_{threshold}-V_{original}\left(r^{3*N}\right)\right)}^{2} \end{array}$$

where *k* is a harmonic force constant.

Both E_*threshold*_ and *k* can be adjusted while their optimal values can be automatically estimated from the regular unbiased MD simulation based on three criteria.

Criterion 1. $V_{boost}\left(r^{3*N}\right)$ must be a monotonic function that guarantees that the relative rank/order of the biased potentials is the same as the relative rank/order of the original unbiased potentials, such that, given any two arbitrary potentials $V_{original\text{\_}1}\left(r^{3*N}\right)$ and $V_{original\text{\_}2}\left(r^{3*N}\right)$ on the original energy surface, if $V_{original\text{\_}1}\left(r^{3*N}\right)<\;V_{original\text{\_}2}\left(r^{3*N}\right)$ the function $V_{boost}\left(r^{3*N}\right)$ must guarantee that $V_{updated\text{\_}1}\left(r^{3*N}\right)<\;V_{updated\text{\_}2}\left(r^{3*N}\right)$.

Criterion 2. The difference between the two updated potential energies must be less than the difference between the two original potential energies, such that $\left|V_{updated_{2}}\left(r^{3*N}\right)-\;V_{updated_{1}}\left(r^{3*N}\right)|<\;|V_{original_{2}}\left(r^{3*N}\right)\;-\;V_{original_{1}}\left(r^{3*N}\right)\right|$. The combination of criteria 1 and 2, and equations (4) and (5) suggest that equation (6) must be valid, which in turn requires that equation (7) must be valid and that 0 ≤ *k*_0_ ≤ 1.

(6) $$\begin{array}{l} V_{\max}\;\leq E_{threshold}\;\leq V_{\min}+\;\frac{1}{k} \end{array}$$

(7) $$\begin{array}{l} k=k_{0}*\frac{1}{V_{\max}-\;V_{\min}\;}\;\leq \;\frac{1}{V_{\max}-\;V_{\min}\;} \end{array}$$

Criterion 3: $V_{boost}\left(r^{3*N}\right)$ must have a narrow, thus a small standard deviation (equation 8) to make the reweighing using cummulant expansion to the second order possible.

(8) $$\begin{array}{l} \sigma_{V_{boost}}=\;k\left(E_{threshold}\;-\;V_{original_{mean}}\;\left(r^{3*N}\right)\right)\sigma_{V_{original}}\;\leq \;\sigma_{0} \end{array}$$

Where *V*_*origina*_*l*__*mean*__ and σ_*V*_*original*__ are the mean and the standard deviation of the unbiased potential energies, σ_*V*_*boost*__ is the standard deviation of the boosted potential energy, and σ_0_ is an upper limit specified by the user.

The *k*_0_ in equation 7 can be computed as $k_{0}=\min\left(1.0,\;\frac{\sigma_{0}}{\sigma_{V_{original}}\;}*\frac{V_{\max}-\;V_{\min}}{V_{\max}-V_{original_{mean}}}\right)$.

### GaMD reweighing using cummulant expansion

The probability along a reaction coordinate, *RC*(**r**), given as *p*′(*RC*), where **r** is the atomic coordinate $r_{1}^{3},r_{2}^{3},\;\ldots ,\;r_{N}^{3}$, from the boost potential $V_{boost}\left(r^{3^{*}N}\right)$ of each frame, *p*′(*RC*) can be reweighted to obtain the probability distribution, *p*(*RC*) of the canonical ensemble as shown in equation 9.

(9) $$\begin{array}{l} p\left(RC_{j}\right)=\;p^{\prime }\left(RC_{j}\right)*\frac{{\langle e^{k_{B}TV_{boost}\left(r^{3*N}\right)}\rangle }_{j}}{\sum_{j=1}^{M}{\langle e^{k_{B}TV_{boost}\left(r^{3*N}\right)}\rangle }_{j}}\;for\;j=1,\;\ldots ,\;M \end{array}$$

The reweighted free energy can then be obtained as $F\left(RC_{j}\right)=\;-\frac{1}{k_{B}T}\ln p\left(RC_{j}\right)$.

### Residue-residue contact

Detail interactions between TorsinA (or TorsinAΔE303) and LULL1 were examined by residue-residue contact analysis wherein an amino acid of TorsinA/TorsinAΔE303 interacts with an amino acid of LULL1 if the distance between their centers of mass is within 6 Å.

### Hydrogen bonds analysis

A hydrogen bond is recorded between two units whenever a hydrogen-bond acceptor (HBA) of one of the units is within 3.0 Å of a hydrogen-bond donor (HBD) of the other unit and the angle formed by HBA-Hydrogen-HBD is greater than or equal to 135°.

## Data availability

The initial structure of TorsinA/TorsinAΔE303 and its activator (LULL1) as well as the nanobody (VHH-BS2) are available in the Protein Data Bank (PDB IDs: 5j1s, 5j1t). Other dataset (such as Trajectories from Molecular Dynamics Simulations, etc.) upon which the results presented in the paper are based can be requested by sending an email to the corresponding author or to Bioinformatics Center at tools@Bioinformatics.Center.

## Author contributions

ES designed the study, carried out the study, analyzed and interpreted the data, and wrote the manuscript.

### Conflict of interest statement

The author declares that the research was conducted in the absence of any commercial or financial relationships that could be construed as a potential conflict of interest.
