# Supplementary material for: The Impairment of TorsinA's Binding to and Interactions With Its Activator: An Atomistic Molecular Dynamics Study of Primary Dystonia
Source: Front Mol Biosci. 2018 Jul 10;5:64. doi: 10.3389/fmolb.2018.00064 (PMC6048259; doi:10.3389/fmolb.2018.00064)
Supplement: Supplementary file 1 [file Table_1.DOCX]

**SUPPLEMENTARY MATERIAL**

Title: **The Impairment of TorsinA's Binding to and Interactions with its Activator: An Atomistic Molecular Dynamics Study of Primary Dystonia**

Short Title: **Disruption of TorsinA-Activator Interactions in Primary Dystonia**

Emmanuel Oluwatobi Salawu ^1,*^

ORCID: 0000-0002-4977-0917
^1^ TIGP Bioinformatics Program, Academia Sinica, Taiwan; Institute of Bioinformatics and Structural Biology, National Tsing Hua University, Taiwan; School of Computer Science, University of Hertfordshire, United Kingdom; Bioinformatics Center, Sheridan, Wyoming 82801, United States; *emmanuel@gapp.nthu.edu.tw

**Supplementary Figure S1.** Residue-residue distances between amino acids of TorsinA/TorsinAΔE303 and those of LULL1

**Supplementary Figure S2.** Hydrogen bonding between TorsinA and LULL1 is highly compromised by ΔE303

**Supplementary Table S1.** VHH-BS2, the nanobody used for the crystallization, attenuates the effects of the mutation (ΔE303) on the overall secondary structure elements compositions of TorsinA and TorsinAΔE303 in addition to reducing the differences between "TorsinA-LULL1 interactions" and "TorsinAΔE303-LULL1 interactions"


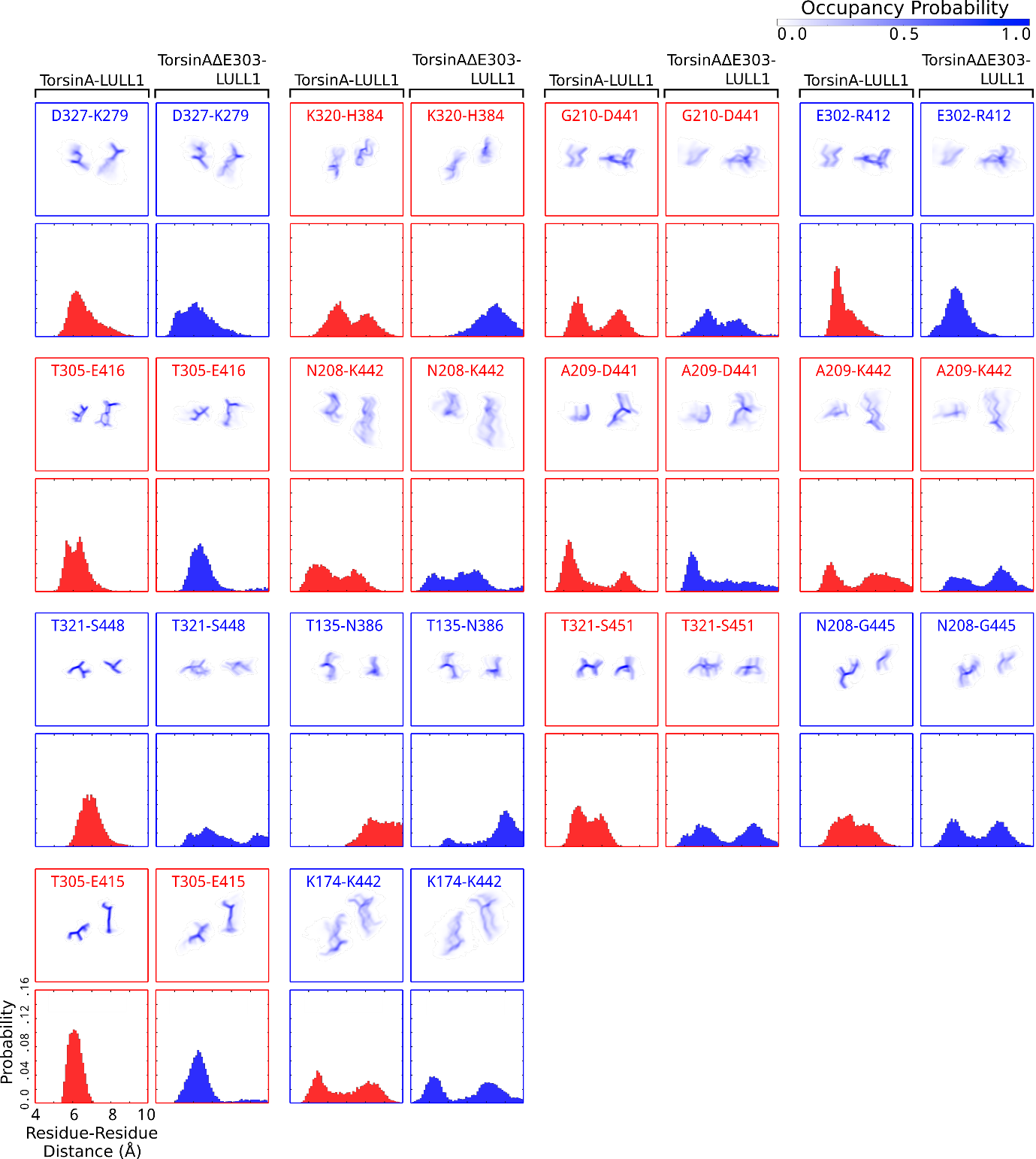


**Supplementary Figure S1. Residue-residue distances between amino acids of TorsinA/TorsinAΔE303 and those of LULL1.** This figure is an extension/a continuation of **Fig. 3** showing detail account of the effects of ΔE303 on residue-residue *("amino acid of TorsinA/TorsinAΔE303"-"amino acid of LULL1" e.g. T135-Y379)* distances. A red bounding-box shows that (for the TorsinA-/TorsinAΔE303-LULL1 residue pair) TorsinA-LULL1 interactions are better than TorsinAΔE303-LULL1 based on a cut-off distance of 6 Å, otherwise a blue bounding-box. In each group of four boxes, the top row shows the distributions of the position of the amino acid of TorsinA-/TorsinAΔE303 and that of LULL1 in form of occupancy probability ranging from 0.0 (white) to 1.0 (dark blue), while the bottom row contains histograms showing the distributions of TorsinA-/TorsinAΔE303-LULL1 distances.


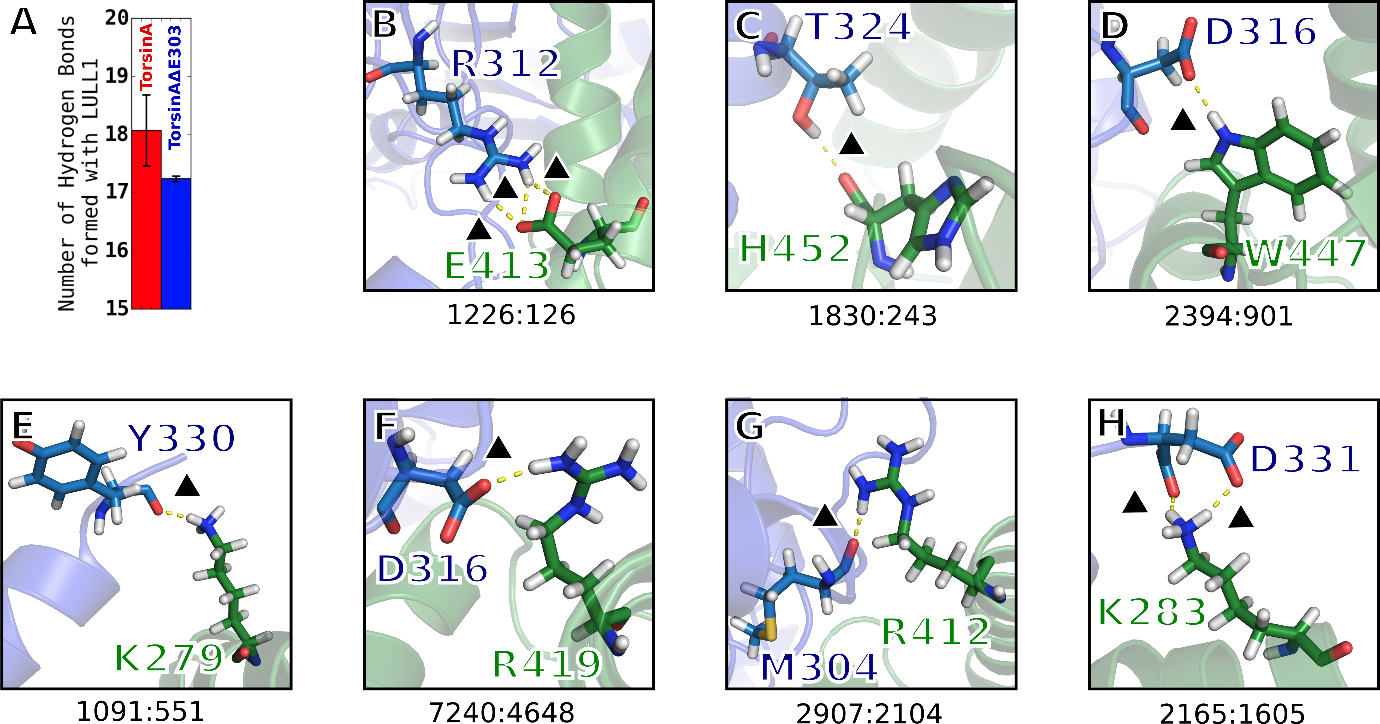


**Supplementary Figure S2. Hydrogen bonding between TorsinA and LULL1 is highly compromised by ΔE303.** This is a continuation of Fig. 5. **(A)** Compared to TorsinA, TorsinAΔE303 forms fewer hydrogen bonds with LULL1. The difference is statistically significant (p < 0.001). Panels **B** to **H** show examples of hydrogen bonds that are frequently seen in TorsinA-LULL1 interactions but are seldom seen in TorsinAΔE303-LULL1 interactions. The hydrogen bonds are represented by yellow broken lines. A black triangle is placed next to each of the hydrogen bonds to guide the readers’ eyes. The amino acids of TorsinA are shown in blue, while those of LULL1 are shown in green. The ratio of the frequency of a hydrogen bonding pattern in TorsinA-LULL1 interactions to its frequency in TorsinAΔE303-LULL1 interactions are presented as the numbers below each panel (e.g. TorsinA-LULL1:TorsinAΔE303-LULL1 = 1226:126 in panel B).

**Supplementary Table S1.** VHH-BS2, the nanobody used for the crystallization, attenuates the effects of the mutation (ΔE303) on the overall secondary structure elements compositions of TorsinA and TorsinAΔE303 in addition to reducing the differences between "TorsinA-LULL1 interactions" and "TorsinAΔE303-LULL1 interactions"

|  | With VHH-BS2, the nanobody used for the crystallization | | Without VHH-BS2, a more physiologically meaningful setting | |
| --- | --- | --- | --- | --- |
|  | Proportion of Helix (%) | Proportion of Sheet (%) | Proportion of Helix (%) | Proportion of Sheet (%) |
| TorsinA | 45.3 | 9.3 | 45.0 | 9.0 |
| TorsinAΔE303 | 45.8 | 9.5 | 44.0 | 9.5 |
| *Change in Proportion due to ΔE303 | 0.5 | 0.2 | 1.0 | 0.5 |

**At least 4500 structures were analysed to obtain each of the secondary structure elements proportions reported here. All the observed changes are statistically significant, and the differences between secondary structures proportions for TorsinA and TorsinAΔE303 are also statistically significant (p < 0.001).*

The raw secondary structures assignments results are available on RaFoSA's web server(Salawu, 2016) at Bioinformatics Center and can be accessed using the following URLs.

TorsinA <VHH-BS2>: <http://bioinformatics.center/servers/rafosa/results/59461904m>
TorsinAΔE303 <VHH-BS2>: <http://bioinformatics.center/servers/rafosa/results/594619e3p>
TorsinA: <http://bioinformatics.center/servers/rafosa/results/59461a1au>
TorsinAΔE303: <http://bioinformatics.center/servers/rafosa/results/59461a3ae>
